# The positional consistency between guidewire and cannulated or solid screw in robot-assisted spinal internal fixation surgery

**DOI:** 10.1186/s13018-023-04053-4

**Published:** 2024-01-05

**Authors:** Jingwei Zhao, Yunxian Zhang, Mingxing Fan, Xiaoguang Han, Bo Liu, Da He, Wei Tian

**Affiliations:** 1 Department of Spine Surgery, Beijing Jishuitan Hospital, Capital Medical University, 31 Xinjiekou East Street, Xicheng District, Beijing, 100035 China; 2 Research Unit of Intelligent Orthopedics, Chinese Academy of Medical Sciences, Beijing, China; 3 School of Biomedical Engineering, Capital Medical University, Beijing, China

**Keywords:** Positional consistency, Guidewire, Screw, Spinal surgery, Robot-assisted, Percutaneous, Image fusion, Intra-operative CT, Post-operative CT, Cone-beam CT

## Abstract

**Background:**

This study aimed to investigate the positional consistency between the guidewire and the screw in spinal internal fixation surgery.

**Methods:**

This study involved 64 patients who underwent robot-assisted thoracic or lumbar pedicle screw fixation surgery. Guidewires were inserted with the assistance of the Tirobot. Either cannulated screws or solid screws were inserted. Guidewire and screw accuracy was measured using CT images based on the Gertzbein and Robbins scale. The positional consistency between guidewire and screw was evaluated based on the fused CT images, which could graphically and quantitatively demonstrate the consistency. The consistency was evaluated based on a grading system that considered the maximum distance and angulation between the centerline of the guidewire and the screw in the region of the pedicle.

**Results:**

A total of 322 screws were placed including 206 cannulated ones and 116 solid ones. Based on the Gertzbein and Robbins scale, 97.5% of the guidewires were grade A, and 94.1% of the screws were grade A. Based on our guidewire-screw consistency scale, 85% in cannulated group, and 69.8% in solid group, were grade A. Both solid and cannulated screws may alter trajectory compared to the guidewires. The positional accuracy and guidewire-screw consistency in the solid screw group is significantly worse than that in the cannulated screw group. The cortical bone of the pedicle has a positive guide effect on either solid or cannulated screws.

**Conclusion:**

The pedicle screws may alter trajectory despite the guidance of the guidewires. Solid screws show worse positional accuracy and guidewire-screw consistency compared with cannulated screws.

*Trial registration* The study was retrospectively registered and approved by our center’s institutional review board.

**Supplementary Information:**

The online version contains supplementary material available at 10.1186/s13018-023-04053-4.

## Background

Orthopedic robots have become regular assistant devices during spinal surgery in many medical centers. They have been shown to improve screw accuracy compared with the free-hand method from lumbar to upper cervical vertebrae, according to previous studies [1–17]. However, there is still an inaccuracy rate of 2–10% reported in the literature [1–7]. To our knowledge, few studies have focused on analyzing the mechanisms behind these inaccuracies.

One orthopedic robot provides the positioning of screw trajectory by automatically steering the guide sleeve of the robot arm under the guidance of the optical navigation system [8, 18]. The procedures afterward include the insertion of the guidewire and the screw. Three types of inaccuracies could be induced throughout the entire process: the error between the screw planning and the position of the guide sleeve, the error between the positions of the guide sleeve and the guidewire, and the error between the positions of the guidewire and the screw. The first error has been widely discussed and reduced by engineers. However, the last two errors were often ignored and rarely analyzed. The reason for this may be the difficulty in acquiring the state of the guidewire stage and the lack of proper image analysis methods [1, 8].

Cannulated screws should be used when we choose a guidewire guided procedure. However, to our knowledge, solid screws were used sometimes though guidewires were inserted under financial limitations because solid screws are usually cheaper. However, the positional relationship between the solid screw and the guidewire remains unclear. This study compared the positional consistency of the guidewire and the screw between cannulated screw and solid screw instrumentation surgery using a novel computed tomography (CT) image comparison method. The conclusion of this study may also applicable to percutaneous pedicle screw instrumentation that follows guidewire insertion under fluoroscopy, without the use of an orthopedic robot.

## Methods

### Aim

To investigate the positional consistency of the guidewire and the screw in cannulated screw and solid screw instrumentation surgery.

### Study design

Retrospective case series. The study involved 64 consecutive patients who underwent robot-assisted thoracic or lumbar pedicle screw fixation surgery in our center from Jan. 2019 to Jan. 2020.

### Surgical procedure

All surgeries were assisted by the Tirobot system (TINAVI, China). The illustrated robot component and typical surgical procedure has been described previously [8, 18]. (1) Robot positioning. After proper exposure, intraoperative CT (IOCT) image acquisition and registration, and screw planning, the robot arm with the guide sleeve steered itself toward the planned screw trajectory. (2) Guidewire insertion. The guide sleeve was inserted onto the bone surface, which would be the entry point of the pedicle screw. Through the guide sleeve, the surgeon inserted the guidewire into the pedicle using an electric drill. The typical insertion depth of the guidewire was 35 mm. (3) Guidewire confirmation. The second intraoperative CT image was acquired to confirm the positional accuracy of the guidewire. (4) Trajectory enlargement. The surgeon used a cannulated cortical breacher and cannulated screw tap to enlarge the trajectory. (5) Screw insertion. When cannulated screw was chosen, it would be inserted along the guidewire. When solid screw was chosen, it would be inserted into the trajectory hole after the guidewire was removed. The intraoperative CTs were acquired using the Orbic-C 3D arm (Siemens, Germany).

### Guidewire and screw accuracy measurement

The accuracy of the guidewires and the screws were evaluated using IOCT and POCT images, respectively. The Gertzbein and Robbins [19] scale was used to assess the accuracy of the guidewires and the screws. Originally, the scale was designed to describe the position of the screws. In this study, we describe the position of the guidewires and the screws similarly as follows: GR-A, the instrumentation locates inside the pedicle; GR-B, the instrumentation breaches the cortical bone of the pedicle for less than 2 mm; GR-C, the instrumentation breaches the cortical bone of the pedicle for equal to or more than 2 mm.

### Image fusion

To assess the positional consistency between the guidewire and the screw, we fused IOCT with postoperative CT (POCT) images. Since the relative position of different vertebrae may change between IOCT and POCT due to reduction, inter-segmental fusion, and rod fixation procedures, we fused and assessed different vertebrae separately.

The image fusion tool used was General Registration (BRAINS) from 3D-Slicer software version 4.13.0 (www.slicer.org) [20]. This tool can fuse two CT images based on their regional similarity, regardless of their different resolution. The target vertebra was regionally marked as the region of interest for fusion to avoid the influence of other vertebrae. 10 percent of image pixels were used for fusion. We used rigid transformation, also known as 6 degrees of freedom (DOF) transformation, during image fusion. The fusion method ran iteratively until single-step improvement was less than 0.1 percent.

**Fig. 1:**
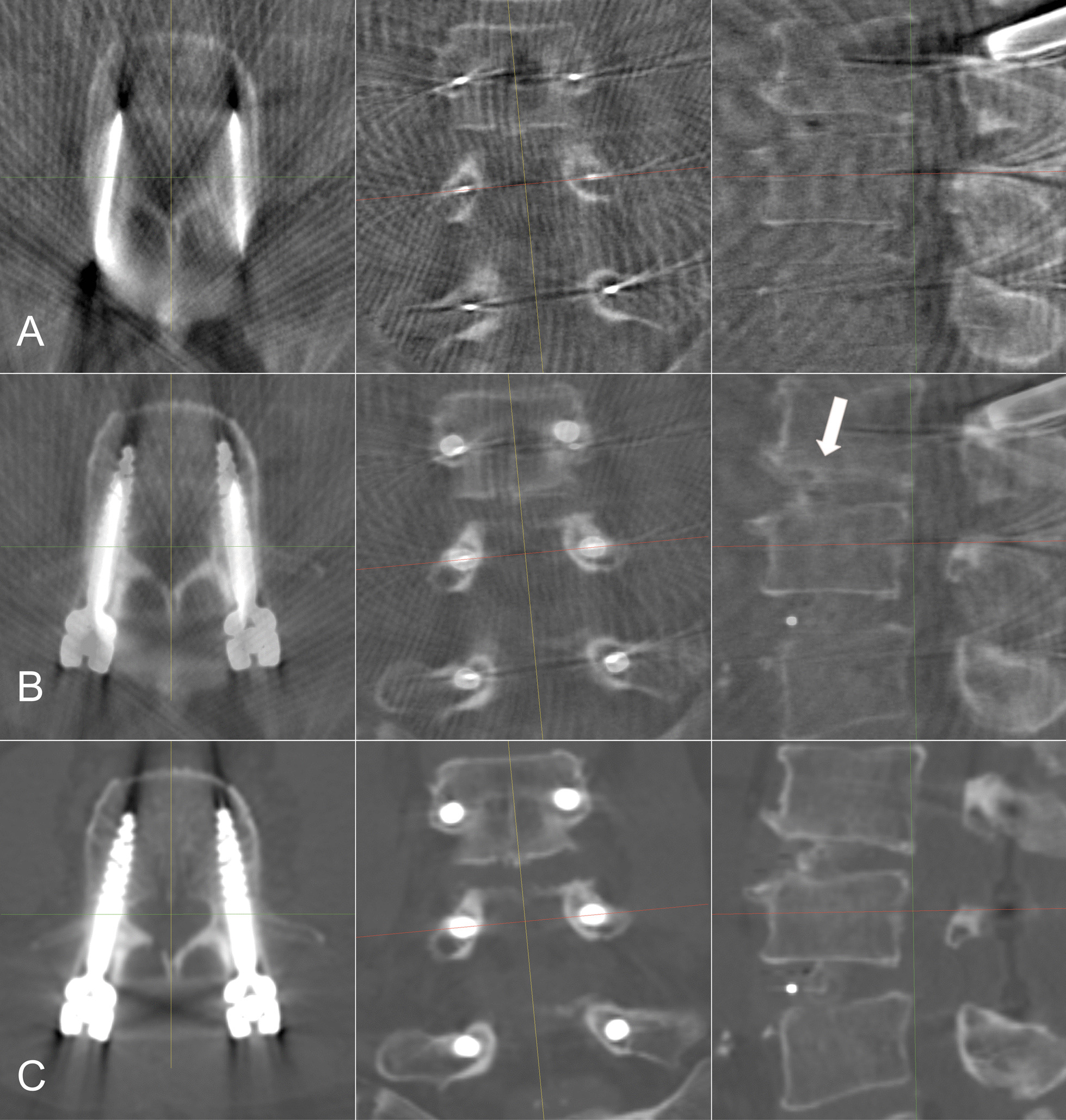
Image fusion result of IOCT and POCT, and assessment of the fusion. Each row shows three perpendicular reconstructed planes from a same CT. **A**: IOCT; **B**: Image overlay of IOCT and POCT. The target vertebra (centered) is fused. Note that the adjacent vertebra (white arrow) cannot be fused simultaneously because of the inter-segmental movement, thus it shows double edges of cortical bone; **C**: POCT

After image fusion, we assessed the fusion result. In multiplanar reconstructions, including axial, coronal, and sagittal views, we used an overlay image of IOCT and POCT to confirm that the vertebral cortical edges overlapped with each other (Fig. 1). As shown in the image, the target vertebra, which was the fourth lumbar vertebra in the case, was successfully fused, while the third lumbar vertebra was apparently not because the inter-segmental cage changed the relative position between the vertebrae. Each vertebra was fused and assessed separately. The bony structures were more distinguishable in dynamic overlap-image series, which were used as the main method to verify the fusion results (Additional file 1, Additional file 2 and Additional file 3).

### Consistency assessment between guidewire and screw

**Fig. 2:**
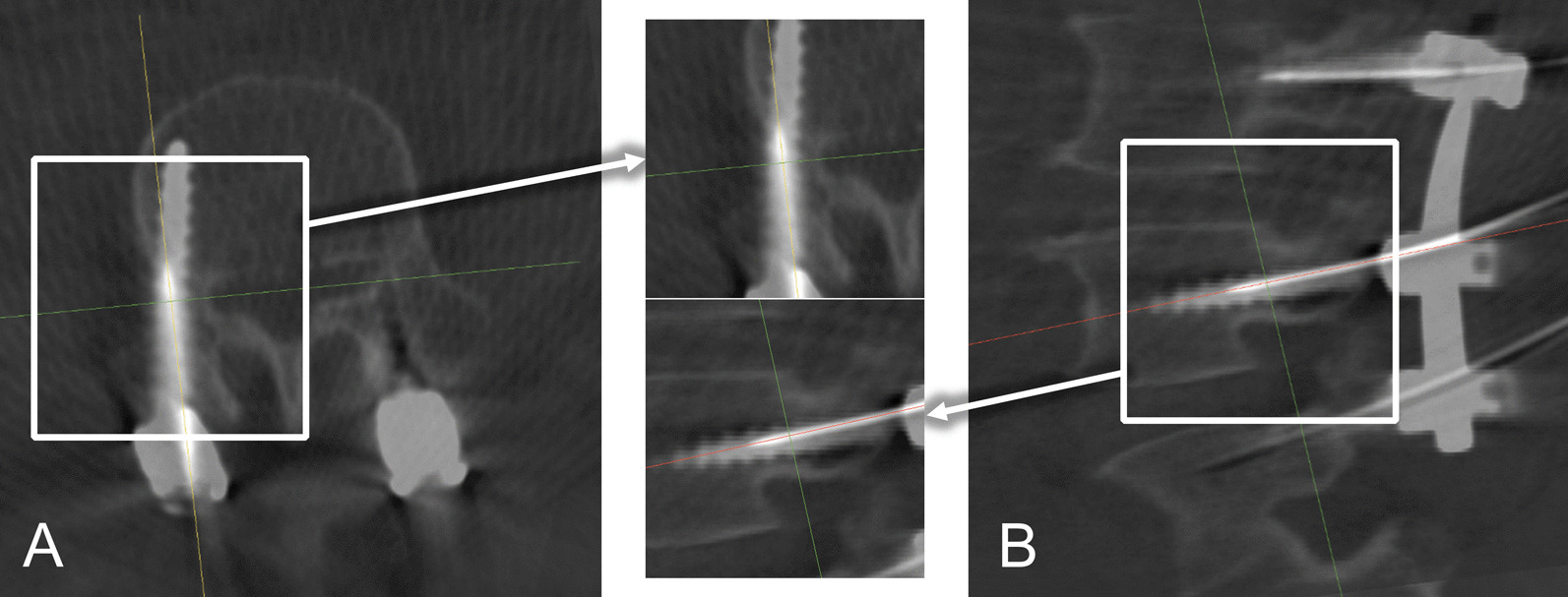
Consistency assessment image of guidewire and screw. Assessment images for one screw as an example. Key regions of axial (**A**) and sagittal (**B**) planes of a same screw were isolated to demonstrate the relationship between the guidewire and screw (center). Yellow or red lines are positioned along the guidewire to improve its recognizability in the fused image. The following figures in this article follow this pattern to demonstrate the relationship between one guidewire and the corresponding screw

We adjusted the transparency weights of the IOCT and the POCT to reveal both the guidewire and the screw in the same fused image. We used an axial plane and a sagittal plane to assess one screw. (Fig. 2) As the image shows, both the guidewire and the screw are recognizable, and the positional and angular differences are measured.

**Fig. 3:**
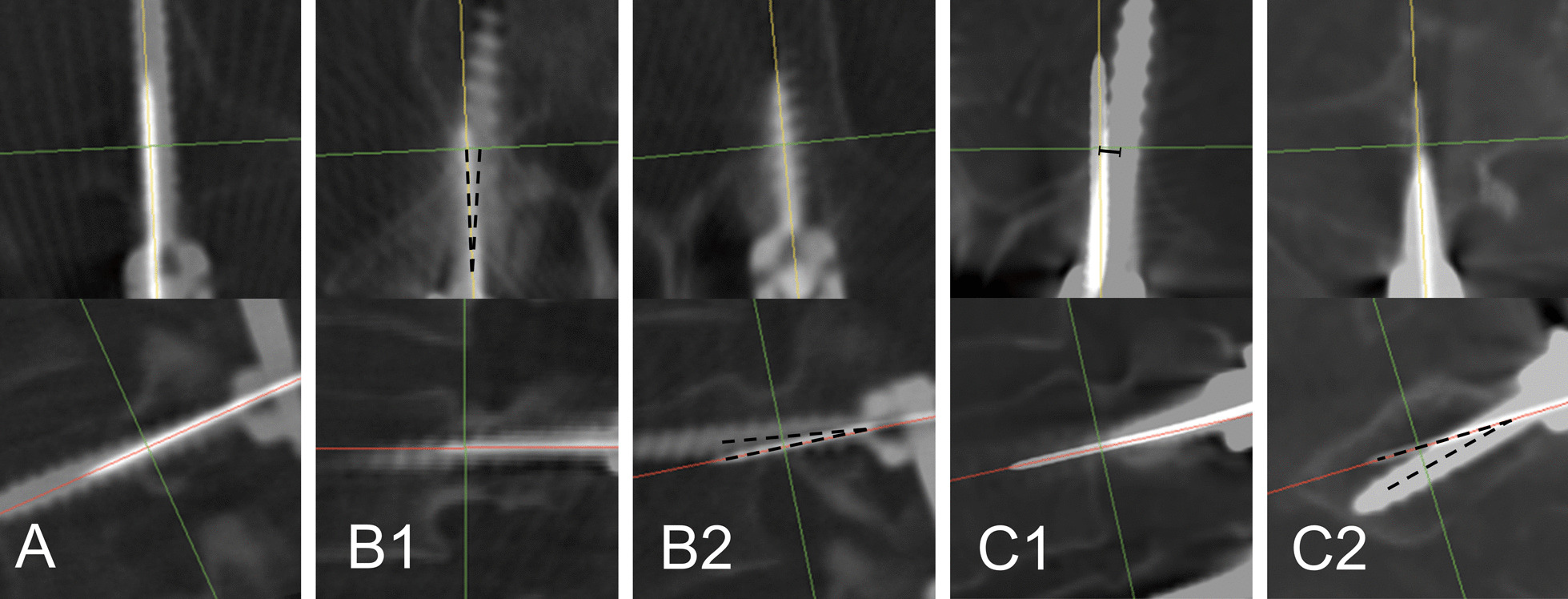
Consistency scale between guidewire and screw.** A**: Grade A, maximum distance between guidewire and screw less than 2 mm and angle less than 5 degrees in the region of pedicle;** B**: Grade B, maximum distance less than 2 mm and angle between 5 (included) and 10 (excluded) degrees. Axial angulation of 6.9° in** B1** and Sagittal angulation of 6.2° in** B2**;** C**: Grade C, maximum distance equal to or more than 2 mm, or angle equal to or more than 10 degrees. Distance of 3.5 mm in** C1** and angulation of 13.7° in** C2**

Since there was no similar reference, we made a novel evaluation scale based on this group of cases. In the region of pedicle, for each screw, we measured the maximum distance and angle between the center line of the wire and screw. Grade A: maximum distance less than 2 mm and angle less than 5 degrees; Grade B: maximum distance less than 2 mm and angle between 5 (included) and 10 (excluded) degrees; Grade C: maximum distance equal to or more than 2 mm, or angle equal to or more than 10 degrees. Worse result was adopted ether in the axial or the sagittal plane. (Fig. 3).

### Statistical analysis

The results were measured by two experienced spinal surgeons, and a consensus was achieved through discussion. All statistical analyses were performed using SPSS 25.0 (IBM, USA). Values are presented as mean ± standard deviation.

## Results

A total of 64 patients with 322 screws were involved in this study. All surgeries were successfully performed under the assistance of the robot. The canulated screw group included 41 patients with 206 screws, and the solid screw group included 23 patients with 116 screws. There were 36 females and 28 males. There were 51 patients who were diagnosed with degenerative diseases and 13 patients with trauma. The mean age was 56.3 ± 12.4 and 50.7 ± 10.0 years in cannulated screw group and solid screw group, respectively. (Table 1).

**Table 1:** Basic information of the patients

	Cannulated screw group	Solid screw group	Sum
Screws (no.)	206	116	322
Diagnosis			
Degenerative disease (no.)	33	18	51
Trauma (no.)	8	5	13
Gender			
Male (no.)	18	10	28
Female (no.)	23	13	36
Age (years)	56.3 ± 12.4	50.7 ± 10.0	/

### Guidewire and screw accuracy

Of the 322 guidewires, 314 (97.5%) were GR-A, and 8 (2.5%) were GR-B. Of the 322 screws, 303 (94.1%) were GR-A, 17 (5.3%) were GR-B, and 2 (0.6%) were GR-C. (Table 2) No revision surgery was required in both groups.

**Table 2:** Guidewire and screw accuracy

Instrumentation accuracy	GR-A	%	GR-B	%	GR-C	%
Guidewires	314	97.5	8	2.5		
All screws	303	94.1	17	5.3	2	0.6
Cannulated screws	198	96.1	8	3.9		
Solid screws	105	90.5	9	7.8	2	1.7

### Positional consistency between the guidewire and the screw

175 (85.0%) of the 206 cannulated screws were Grade A, while, 81 (69.8%) of the 116 solid screws were Grade A according to the consistency scale. Table 3 shows the consistency scale between the guidewire and the screw in the cannulated screw group and the solid screw group. The consistency in the solid screw group was significantly worse than that in the cannulated screw group (Chi-square test *P* < 0.01).

**Table 3:** The consistency between the positions of the guidewires and the screws

Consistency scale	Cannulated screw group	%	Solid screw group	%	p*
Grade A	175	85.0	81	69.8	< 0.01
Grade B	29	14.1	22	19.0	
Grade C	2	1.0	13	11.2	

*Chi-square test

## Discussion

Robot-assisted pedicle screw instrumentation has become a standard technique in many medical centers due to its proven accuracy and safety, especially when compared to the free-hand method. However, a few screws, reportedly 2–10%, are not ideally placed. Some studies have revealed this fact, however none of them have focused on the procedure after the placement of the guidewire. In fact, the orthopaedic robot system can only provide accurate positioning before the placement of the guidewire by automatically steer the guide sleeve along the target trajectory. Procedures that follow, including guidewire insertion, cortical breaching, trajectory tapping, and screw insertion, are all free-hand maneuvers that may introduce errors.

This study compared the position of the guidewires and screws in pairs. Rather than using the Gertzbein and Robbins [19] scale to calculate the overall rate of inaccuracy, this study was able to measure the consistency quantitatively between the wires and screws, as well as analyze the inaccurate cases graphically, based on the precise image fusion method. Besides the method described in this article, the consistency could also be assessed by recording the coordinates of the guidewires and screws and calculating the distances and angulations. However, the method in this article can demonstrate an intuitive graphical view of the consistency, and the grading system is sufficiently accurate and facilitates distinguishing the differences between the wires and screws.

**Fig. 4:**
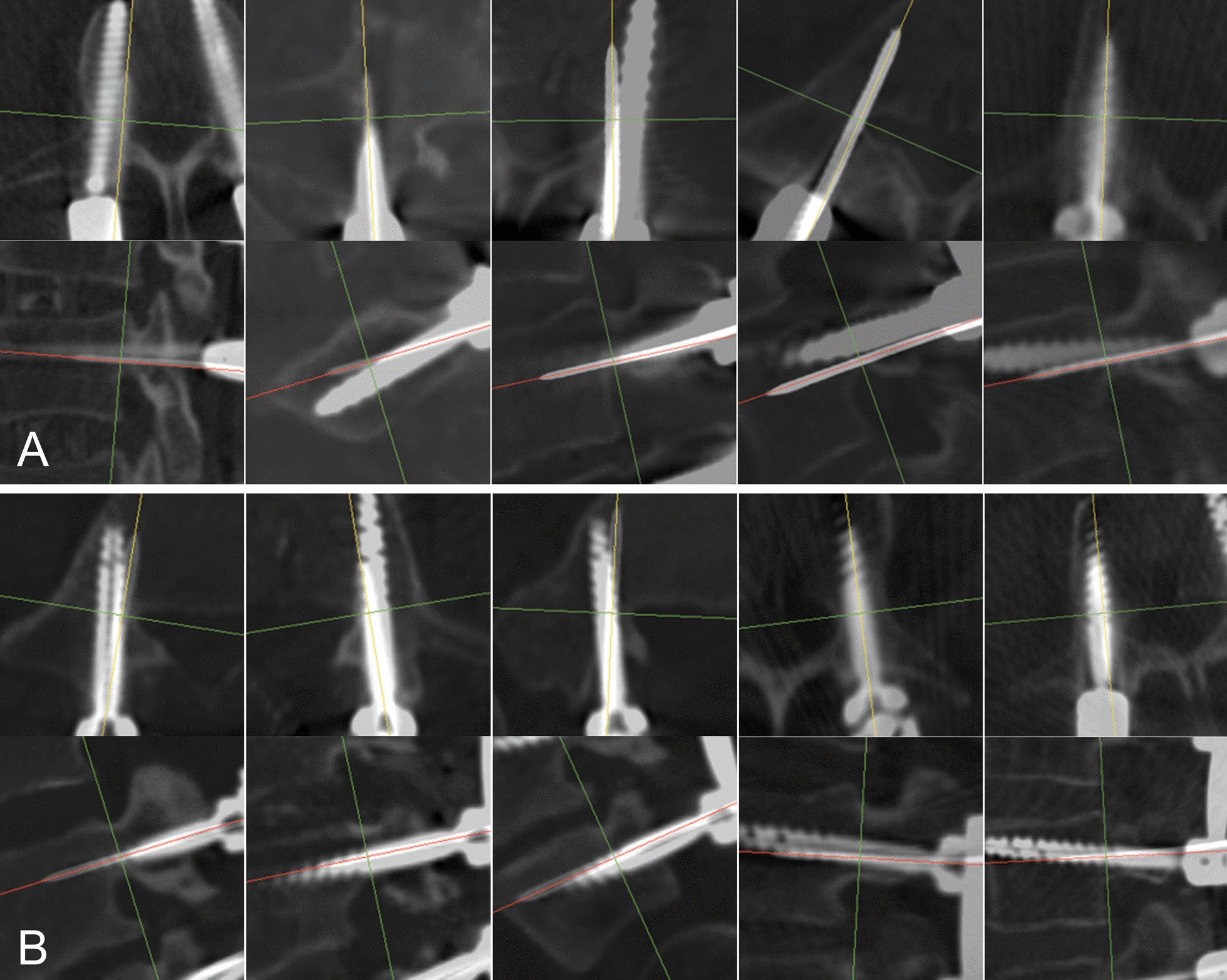
Examples of low grade cases with respect to the consistency between guidewire and screw. Figure shows the low consistency grade cases in each group with respect to the consistency between guidewire and screw. **A**: Solid screws; **B**: Cannulated screws. Solid screws can alter their trajectory considerably without the guidance of guidewire

Solid screws are not as accurate as cannulated ones. Though the cortical breaching and trajectory tapping procedures were guided by the guidewire, the solid screws could alter their trajectory after the guidewires were removed. (Fig. 4) Solid screws are more susceptible to errors from free-hand maneuvers. It can also be affected by the traction of tight muscle, or be pushed away by hard cortical bone of the pedicle. Although solid screws may be chosen occasionally due to financial or instrumental reasons, they are not ideal choices for robot-assisted spinal surgery. According to this study, caution must be taken when choosing solid screws to prevent severe alteration of the trajectory.

**Fig. 5:**
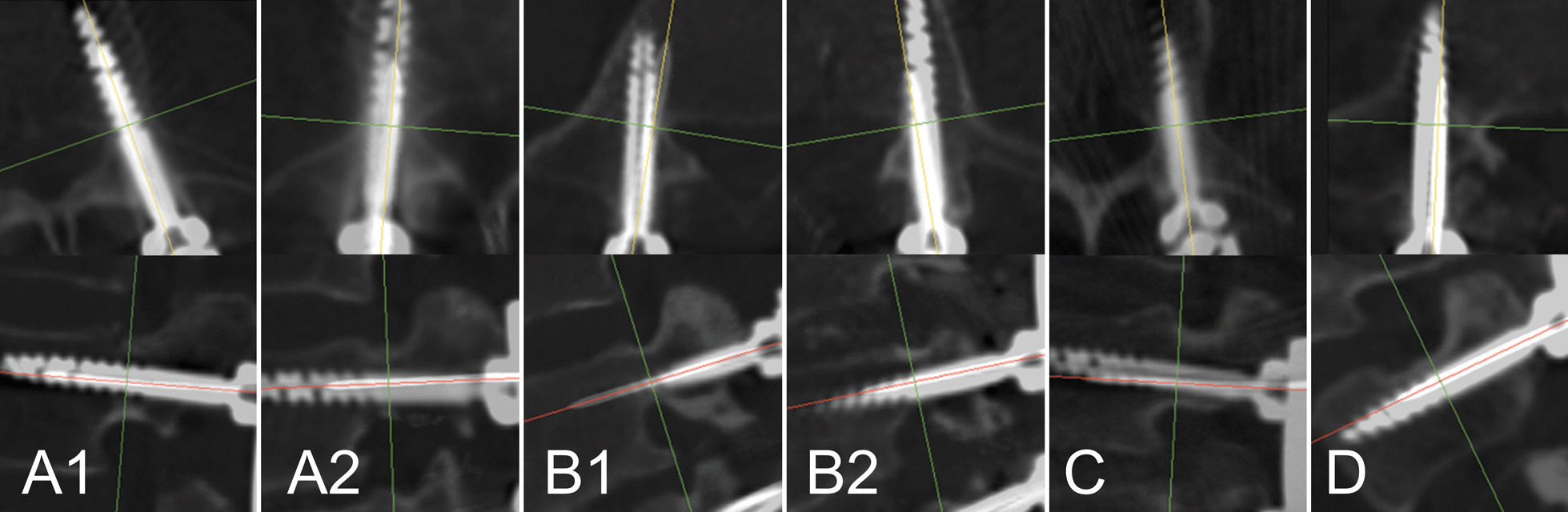
Guide effect from the pedicle to the screw. **A**: Typical guidewires and screws show good consistency; **B**–**D**: Screw was pushed away by the medial (**B**), inferior (**C**) or superior (**D**) wall of the pedicle, positively adjusting the screws from marginally safe guidewires to more favorable and safe positions

The cannulated screws could also change direction during wire-guided insertion. When a guidewire was close to the cortical bone of the pedicle, the screw would change the direction rather than breach the cortical bone. The positive guide effect from the pedicle to either solid or cannulated screw is generally believed by surgeons, and this study is the first to reveal it graphically to our knowledge. (Fig. 5).

The study relied on image fusion between CT image sets. Since the inevitable image distortion, mainly from the Cone-beam IOCT, it would not be possible to match the two CT image sets perfectly through a 6-DOF rigid transforming method. An elastic transforming method may lead to a better fusion result, however, this method would make the measurement difficult and unpredictable. In addition, there was no standard to assess the quality of the fusion result. We gradually changed the transparency of the overlay images of the two fused CTs on different reconstructed planes to assess the positions of key bony marks such as the edges of the vertebrae and the facet processes (Additional file 1, Additional file 2 and Additional file 3). Empirically, a maximum of 1 mm mismatch of the edges was accepted. At first, we tried to fuse two or more vertebrae in one fusion, however, the result was not acceptable because of the intervertebral movement between the supine POCT and prone IOCT. The result of single vertebra fusion was stable and satisfying.

**Fig. 6:**
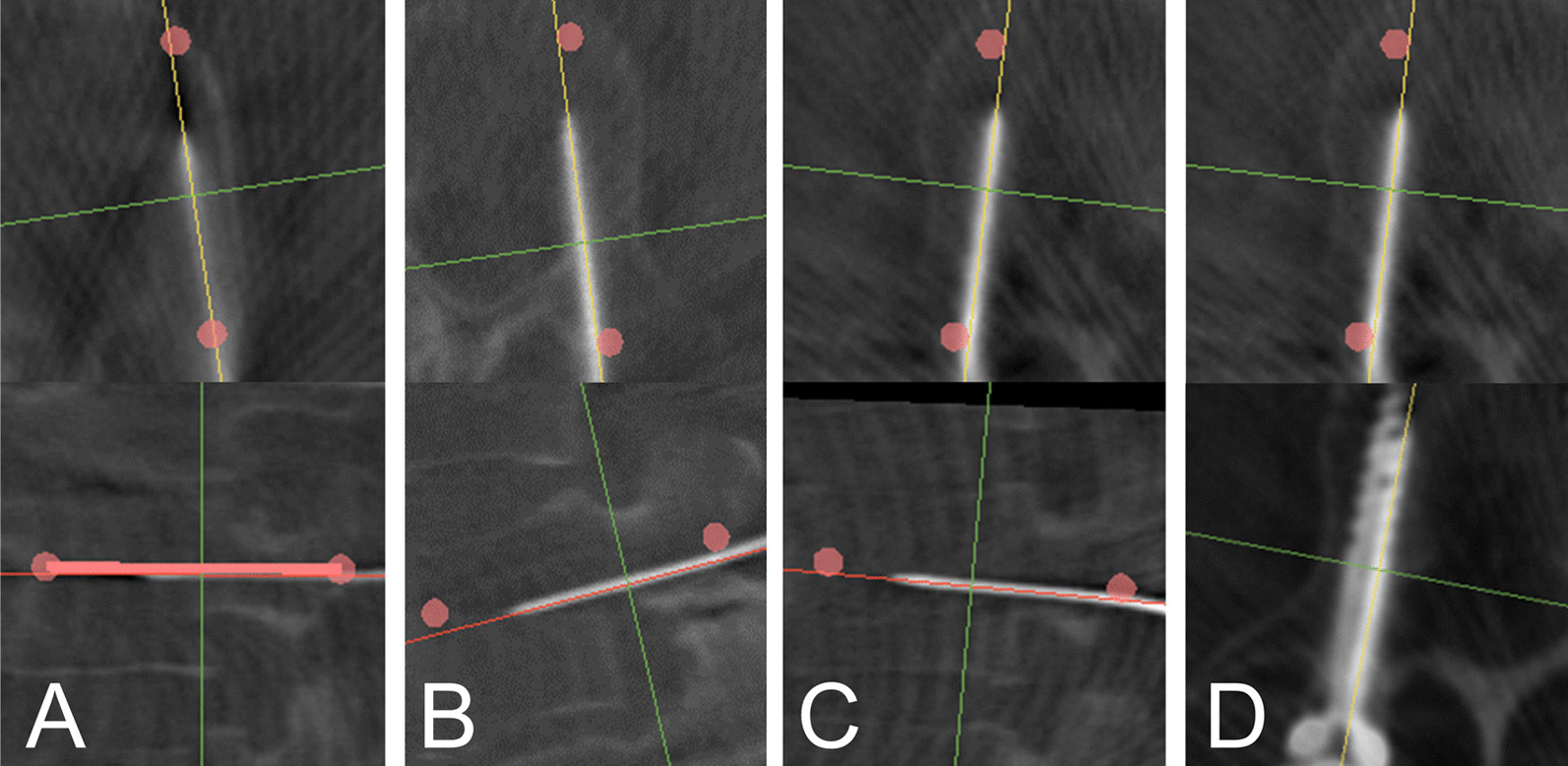
Positional consistency between planning and guidewire/screw. The planning is demonstrated as two red dots indicating the entry point and the end point of the screw. The diameter of the dots is 2.5 mm. The positions of the guidewires could be accurate (**A**) or deviated (**B**,** C**). A deviated guidewire can result in an accurate screw when compared to the planning, under the positive guide effect from the pedicle wall (**D**)

The accuracy of the guidewires and the screws were also reported in this study. The accuracy of the screws (94.1% in GR-A) was consistent with studies using the same robot [1, 8], and the accuracy of the guidewires, firstly reported in this study, was higher (97.5% in GR-A). However, from the graphical demonstration (Fig. 4, 5), some of the guidewires were placed close to the cortical bone of the pedicle, which deviated from their planning. Two main factors may contribute to this: robot positioning error and guidewire sliding at the tangential insertion site. We have developed a method to demonstrate the positional relationship between the screw planning and the guidewire, and preliminary result showed that guidewires may deviate from their planning. (Fig. 6) The positive guide effect of the pedicle could make the screw more consistent with the planning compared with the guidewire. (Fig. 6-D) Further investigation is required to reveal the relationship between the planning and the guidewire/screw and to find out the mechanisms and risk factors affecting the inconsistency.

## Conclusions

The pedicle screws may alter trajectory under the guidance of the guidewires. Solid screws show worse positional accuracy and guidewire-screw consistency compared with cannulated screws.

### Supplementary Information

**Additional file 1**. Image fusion result of IOCT and POCT based on the first vertebra.

**Additional file 2**. Image fusion result of IOCT and POCT based on the second vertebra.

**Additional file 3**. Image fusion result of IOCT and POCT based on the third vertebra.

## Data Availability

The datasets analyzed during the current study are available from the corresponding author on reasonable request.
