# Supplementary figures and images for: The positional consistency between guidewire and cannulated or solid screw in robot-assisted spinal internal fixation surgery
Source: J Orthop Surg Res. 2024 Jan 5;18:708. doi: 10.1186/s13018-023-04053-4 (PMC10768200; doi:10.1186/s13018-023-04053-4)

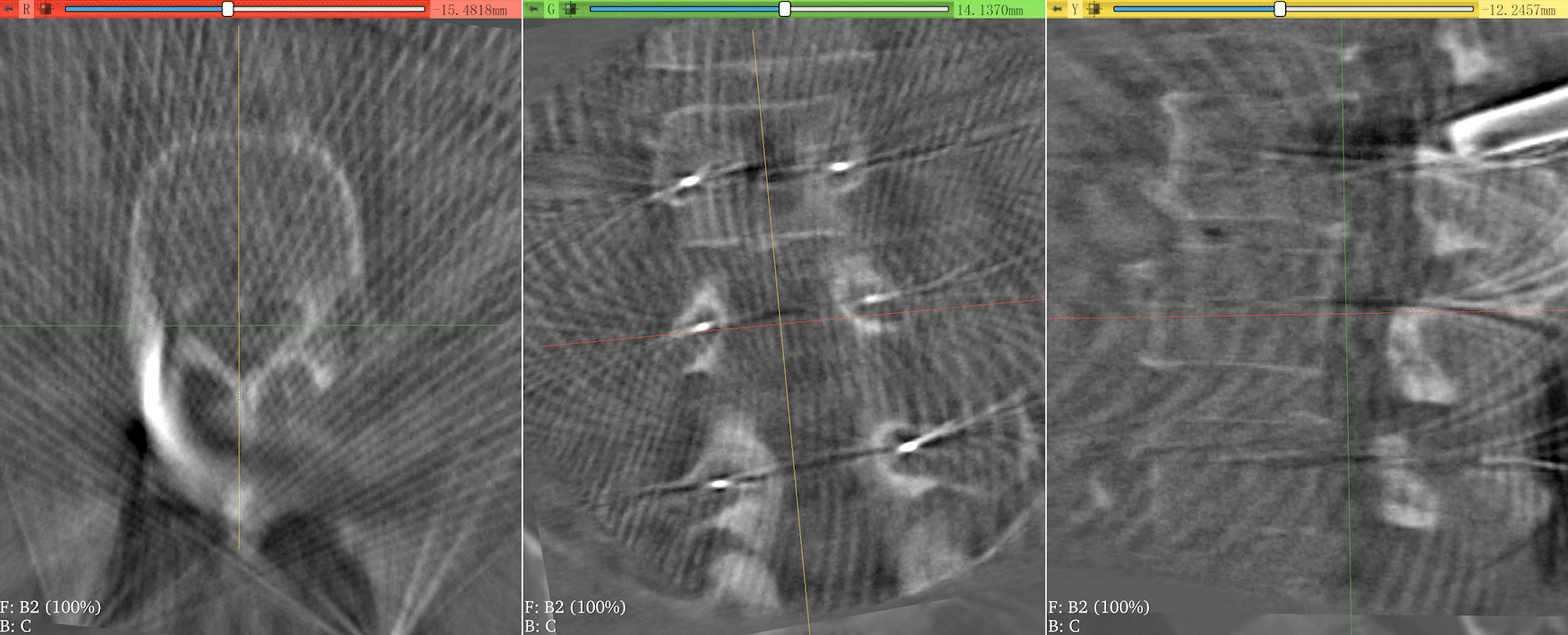

Supplement: Supplementary file 1 — Additional file 1. Image fusion result of IOCT and POCT based on the first vertebra. [file 13018_2023_4053_MOESM1_ESM.gif]

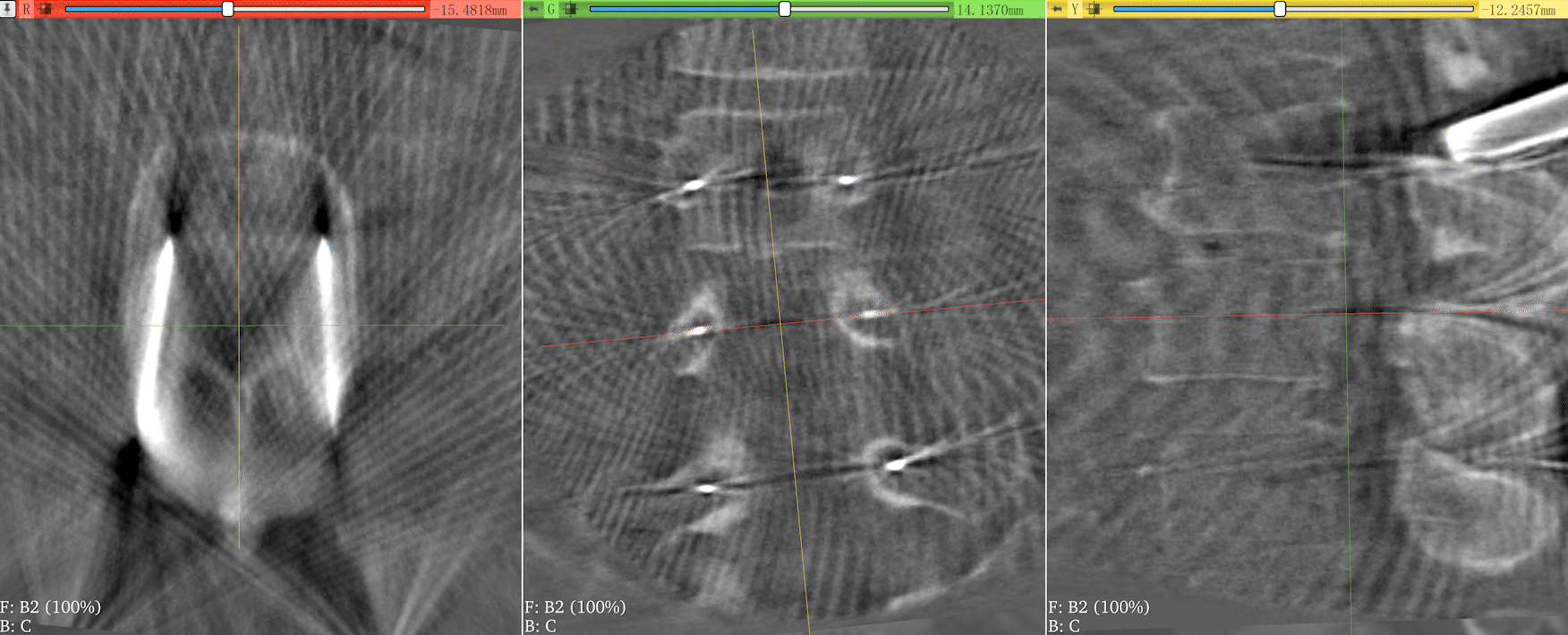

Supplement: Supplementary file 2 — Additional file 2. Image fusion result of IOCT and POCT based on the second vertebra. [file 13018_2023_4053_MOESM2_ESM.gif]

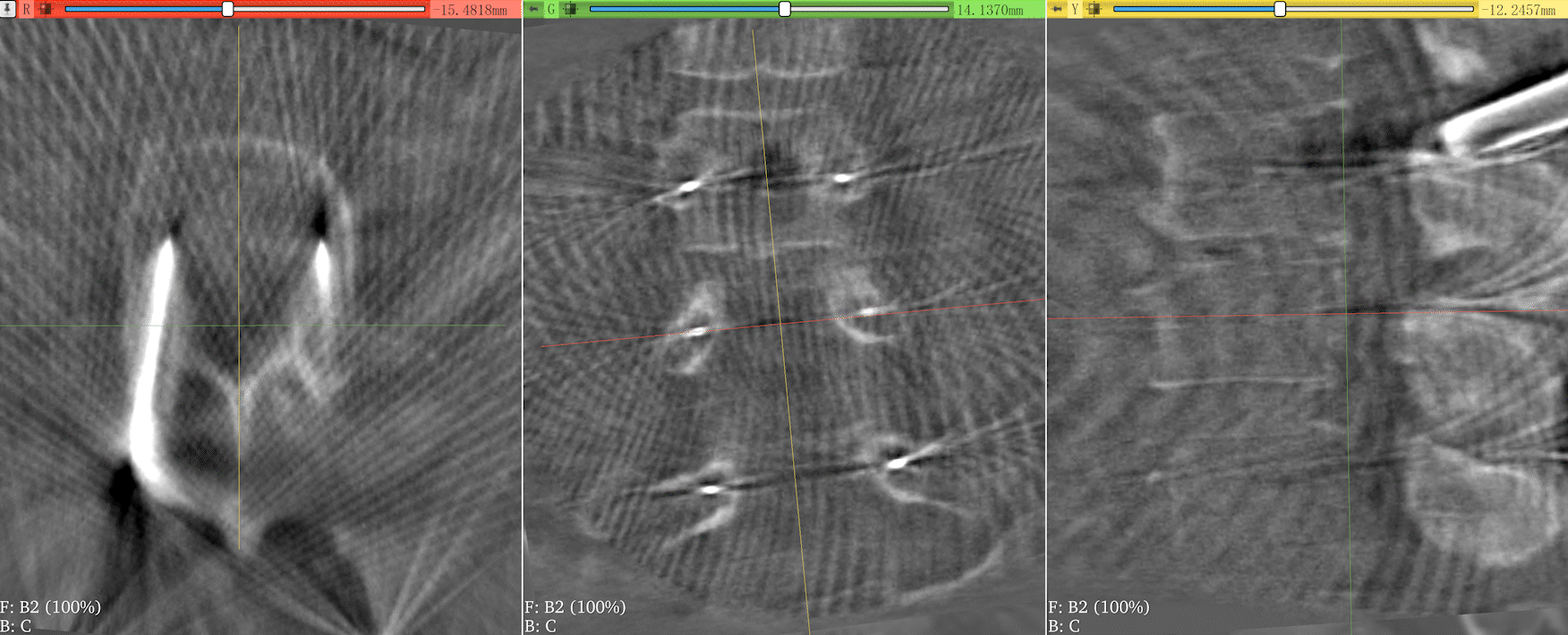

Supplement: Supplementary file 3 — Additional file 3. Image fusion result of IOCT and POCT based on the third vertebra. [file 13018_2023_4053_MOESM3_ESM.gif]
